# Analysis of Electrical Resistance and Impedance Change of Magnetorheological Gels with DC and AC Voltage for Magnetometer Application

**DOI:** 10.3390/s19112510

**Published:** 2019-05-31

**Authors:** Jae-Eun Park, Ga-Eun Yun, Dae-Ik Jang, Young-Keun Kim

**Affiliations:** 1 School of Mechanical and Control Engineering, Handong Global University, Pohang 37554, Korea; jep1009@hanmail.net (J.-E.P.); 21400484@handong.edu (G.-E.Y.); 2 Department Civil and Environmental Engineering, KAIST, Daejeon 34141, Korea; svs2002@kaist.ac.kr

**Keywords:** magnetorheological gel, self-sensing, resistance change, magnetic field sensor, smart material

## Abstract

Magnetorheological gel (MRG) is a smart material that can change its stiffness property by external magnetic field and has been applied as a smart rubber in suppressing vibration. Recent studies show that the electrical resistance of MRG also can be affected with external magnetic field. Thus, this study aimed to conduct analysis on MRG resistance variation due to external magnetic field with DC and AC input voltage. With an DC input voltage, the resistance change due to magnetic field was modeled. In addition, the capacitance variation of the material was observed. The impedance of MRG due to AC input voltage was analyzed and was observed that the impedance of MRG was affected by both the magnetic field and the input frequency. With the experiment data, the impedance modeling of MRG in frequency domain was derived. Based on experiment results, the performance and limitation of MRG as a magnetometer sensor are discussed.

## 1. Introduction

Magnetorheological elastomers (MREs) and magnetorheological gels (MRGs) are smart materials with rheological properties that change depending on the strength of the externally applied magnetic field [1]. MREs are manufactured by mixing iron particles with a silicon or rubber base, whereas MRGs are composed of iron particles in gelatin-based materials [2,3]. An external magnetic field is applied to the magnetorheological (MR) materials during the fabrication process to form chain structures of iron particles aligned along the direction of the magnetic field. The internal stress among the chain structures of iron particles is the main factor that causes changes in the viscoelastic properties of MR materials, and this phenomenon is known as the MR effect (MR effect) [4,5,6,7,8].

Most research on MR materials focuses on the development of vibration suppression systems using the MR stiffness effect. Deng et al. [9] developed a tunable vibration absorber (TVA) made of MREs that increases in stiffness by 118% when a current of 1.5 A is applied to the electromagnet. Komatsuzaki et al. [10] proposed a TVA with a tuning range of 25.8–37.4 Hz under a magnetic field of 316 mT and a current of 6 A. Kim et al. [7] designed an MRE TVA for a linear compressor. The system frequency response varies from 32 to 60 Hz as the stiffness of the MRE changes by 252%, which is achieved under a magnetic field of 240 mT. Recently, MRGs have gained considerable attention because greater variations in stiffness and damping can be achieved in MREs under the same magnetic field. Recently, MRG has gain high attention as it has a wider stiffness and damping variation than MREs under the same magnetic field [4,5,6,7,8,11].

It is possible to design a TVA that has a wider tuning range by using MRGs with a larger variation in stiffness in replacement of MREs. Shin et al. [1] designed an MRG-based TVA with a stiffness change of 380% and a tuning range of 34.7–74.5 Hz under a magnetic field of 200 mT. Kim et al. [12] proposed a hardware design and control algorithm for TVAs based on MRGs. They designed a magnetic field generator based on the principle of the magnetic shield effect and changed the stiffness of an MRG from 25.4 to 36.7 kN/m by controlling the magnitude of the magnetic field. These values correspond to a 44% increase in stiffness under a 100 mT magnetic field, and, consequently, the MRG absorber system has a tunable range of 56–67 Hz.

Besides the stiffness variation, recent studies have shown that the electric resistance of MR materials is also affected by external factors such as the magnetic field and strain change [13,14]. Preliminary studies have shown that the electric resistance of MREs decreases as the magnitude of the magnetic field density and the compression force on the MREs increases [13]. Wang et al. [15] investigated the impedance change of an MRE placed between two steel electrodes. They measured the impedance of the MRE when a sinusoidal voltage signal in the range of 1 Hz to 1 MHz was applied to the electric plates. In that study, the magnitude and the phase angle of the impedance decrease as the magnitude of the magnetic field increases. The effect of the magnetic field on the magnitude of the impedance variation is greater in the low frequency region than the high frequency region. To obtain a better understanding of the impedance of the MRE, an equivalent circuit model, consisting of parallel resistances and capacitances, is proposed.

Using the properties of the impedance change of the MRE by an external magnetic field, MREs have been studied as sensors for a vibration absorber system. Komatsuzaki et al. [10,16] developed a dynamic TVA based on MREs. They observed that change in the MRE resistance is dependent on the frequency of the strain resulting from vibration. Using this phenomenon, the external disturbance frequency was estimated from the MRE resistance without using an external sensor. Sun et al. [17] designed another TVA with a MRE sensor. A laminated steel MRE structure is designed and the frequency of the vibration is estimated from the detected induced voltage in the coils that are wound around the MRE sample.

Electric resistance property of MRGs is studied recently. Yu et al. [18] investigated the effect of various factors, such as the magnetic flux density and carbonyl iron particle (CIP) concentration, on the electric resistance of the MRG. As the magnetic field density increases from 0.1 to 1 T, the resistance of the MRG, composed of 70 wt % CIPs in a polyurethane matrix, decreases from 7.56 to 2.44 MΩ. That research also shows that, as the CIP ratio increases, the resistance of the MRG also increases. In addition, the hysteresis of the MRG is also investigated [18].

MRGs with resistance change can be applied as self-sensing materials for vibration suppression, which can be used to replace sensors such as accelerometers and magnetometers. MRGs are highly nonlinear materials, and the electric resistance is dependent on multiple factors besides the magnetic flux and CIP ratio. Thus, further analysis of the self-sensing properties of MRGs is required. As there are only a few studies on MRG self-sensing, further research on the nonlinear behavior of the MRG sensing properties is necessary to realize engineering applications of these materials.

Therefore, the aim of this research was to analyze the electrical properties of hydrophilic MRGs to be applied as a magnetometer sensor for a tunable vibration absorber, which used the MRG as the stiffness changer [12]. An experiment setup was designed to analyze the resistance and impedance changes in MRGs caused by various factors such as CIP structures, magnetic flux density, time, and frequency of the input voltage. In addition, the settling time and hysteresis of the MRG resistance were analyzed. In addition, the MRG impedance was analyzed in frequency domain to derive the MRG sensing modeling in terms of resistance and capacitance under a certain frequency of AC voltage. Based on the experiment results, the performance and limitation of using the MRGs as self-sensing vibration suppression materials in vibration absorbers are discussed.

This paper is organized as follows. In Section 2, MRG sample preparation and experimental setup are described. In Section 3, the analysis of the changes of electrical resistance and capacitance of MRG due to external magnetic field with DC voltage input is presented. In Section 4, the effect on electrical impedance of the MRG due to AC input voltage frequency and external magnetic field are considered and the electrical system modeling is derived. Based on the experimental results, potentials and limitations of MRG as a magnetic field sensor are discussed.

## 2. Experimental Methods

Experiment setups were designed to examine the self-sensing properties of MRGs. Experiments were conducted to analyze the electrical of DC voltage, impedance of AC voltage, and hysteresis properties of MRGs under various external factors such as magnetic field, input voltage level, and voltage signal frequency.

### 2.1. MRG Sample Preparation

The MRG samples in this work were manufactured using a hydrophilic gelatin base, which is the same material used in our previous paper [1]. The gelatin base was prepared by mixing 1 wt % of carrageenan(No. 34399, MSC CO., LTD, Yangsan, Korea) in water. The carrageenan solution was heated and then 50 wt % carbonyl iron particles (CIP 3189, BASF, Ludwigshafen, Germany) of size 5 μm was added. After stirring the mixture thoroughly, bubbles trapped in the gelatin solution were removed using a vacuum pump. The mixture was then poured into a mold with square base of 32 mm and a thickness of 30 mm. Finally, the mixture was cooled in a refrigerator for more than 12 h to solidify.

Two electrodes of non-magnetic copper were attached on both ends of the MRG sample. Electric wires were connected on the electrodes and to resistors and voltage source to pass current through the sample. In addition, a plastic casing was used to protect the MRG sample from environment factors and being torn out by a strong magnetic field.

### 2.2. Experimental Setup

**Figure 1:**
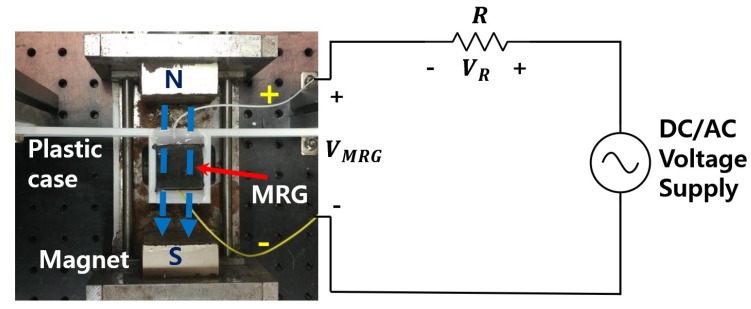
Schematic and photograph of experimental setup.

An experimental setup was designed to observe the MRG’s electrical response according to the change in the magnetic field and input voltage source, as shown in Figure 1. The electric wires on the electrodes of MRG were connected to a 2 kΩ resistor in series, which has similar magnitude of MRG nominal resistance. An electric current was supplied to the MRG sample by applying 1 V DC from a power source, and the voltage across each elements were measured using a DAQ(NI USB-6353) to obtain the resistance of MRG.

The resistance of MRG can be calculated by using the following equation,

(1) $$R_{MRG}\left[k\Omega \right]=2\left[k\Omega \right]\times \frac{V_{MRG}\left[V\right]}{V_{R}\left[V\right]}$$

where $R_{MRG}$ is the resistance of the MRG, $V_{MRG}$ and $V_{R}$ are the voltages of the MRG and the 2 kΩ resistor, respectively.

The impedance of the MRG sample can be measured using the similar manner by applying AC voltage by the power supply.

The magnetic field was applied to the MRG by placing two permanent magnets on either side of the MRG sample. The direction of the magnetic field was set to be parallel to the direction of the CIP chain structures. By adjusting the position of the permanent magnets, the intensity of the magnetic field could be controlled from 0 to 250 mT.

Using the designed setup, experiments were conducted to analyze properties of MRG electrical resistance with respect to the following external factors: (1) CIP chain structure; (2) magnetic field; (3) DC voltage input; and (4) AC input voltage.

## 3. Variation of Electrical Resistance with DC Input Voltage and Magnetic Field

### 3.1. Effect of Chain Structure on MRG Resistance Change

Experiments were conducted to determine the effect of CIP distribution on the resistance characteristics of MRGs. Two types of MRG were prepared for this purpose. The first MRG sample was iostropic, which was cured without applying a magnetic field to obtain a random distribution of iron particles. The second sample was an anisotropic MRG, which was magnetized at 150 mT during the gelatin solidification process to form CIP chain structures within the base matrix. The resistance change of these different types of MRG was then compared to analyze the effects of chain structures on the material sensing properties.

**Figure 2:**
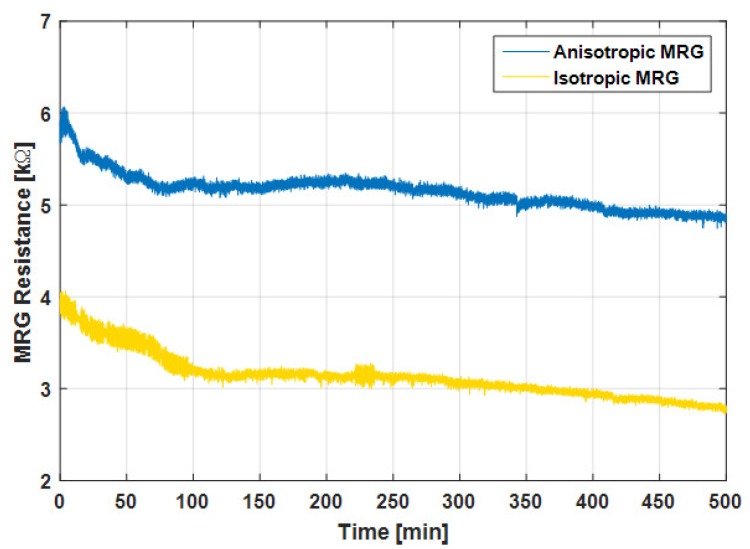
Change in resistance of isotropic and anisotropic MRG over time under 0 mT.

A step input of 1 V DC voltage was applied to the experimental circuit and the resistance changes of the two MRG samples under no magnetic field were measured, as shown in Figure 2.

The resistance of the isotropic MRG sample decreased slightly and reached a steady state value of about 3.2 kΩ after 100 min. The resistance of the anisotropic MRG also reached a steady value of about 5.2 kΩ in about 70 min, faster than isotropic sample. The result shows that a certain amount of time was required for the resistance to reach a steady state when a step input of voltage was applied to the MRG, and experiments were conducted after waiting for this settling time. Since the anisotropic chain structure showed a shorter time for stabilization, the MRG with aligned CIP structures was used as the test specimen throughout this study.

### 3.2. Effect of Magnetic Field on MRG Resistance under DC Input Voltage

The effect of the magnetic field on the resistance of MRG was investigated by applying a DC voltage of 1 V under various external magnetic field. For this test, an anisotropic aqua-based MRG sample was manufactured with 50 wt % CIPs and was magnetized under 150 mT during curing. The change of MRG resistance was measured by comparing the final resistance at the steady state with the initial resistance value of without magnetic field. To obtain a steady value of initial resistance, a constant DC voltage of 1 V was applied to the MRG sample for approximately 1 h prior to the test. The change of MRG resistance was then measured under various magnetic field densities of 0, 50, 100, 150, and 250 mT.

**Figure 3:**
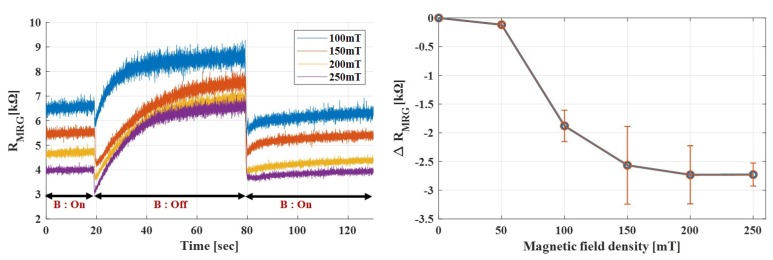
Variation in MRG resistance with magnetic flux density. Resistance decreases with increasing magnetic field density. Right sided figure shows the overall change of resistance for each magnetic field density.

As shown in Figure 3, the MRG resistance decreased when the magnetic field density was increased. As the magnetic field density increased from 0 to 250 mT, the MRG resistance decreased constantly. The changes of resistance due to magnetic field is known as magnetoresistance.

When a voltage was applied to the MRG, the current could flow through the CIPs and water solution, both of which can conduct electricity. Since the tested MRG sample was anisotropic, which contained aligned CIP chain structures, there was an internal path for electric charges to flow from one electrical plate to the other plate, which were placed on both ends of MRG. As the magnetic field was applied to the MRG in the direction of the chains, the embedded carbonyl particles were forced to align in a straighter chain structure that made the electric current to flow more easily through the MRG sample. The gaps between the iron particles in the matrix became smaller when magnetic field was applied as the internal carbonyl iron particles tended to be aligned in more chain structures along the magnetic field direction. This consequently brought the particles to be closer to one another, which led to a reduction in the overall resistance.

Note that, unlike the CIPs in MREs, which were at fixed positions, the CIPs in MRGs were more free to move through the weak gelatin matrix. Thus, a stronger magnetic field caused CIPs to position in more chain structures, which was the main reason for the overall resistance reduction.

However, as there was a limitation to the alignment of the chain structures, the effect of the magnetic field density would reach saturation beyond a certain intensity. This experiment could not be conducted under magnetic field higher than 250 mT, as it would have physically tore the soft MRG at a high level of magnetic field. In summary, the applied magnetic field could be measured by using the estimated function of resistance change with respect to applied magnetic field, as shown in Figure 3.

### 3.3. Effect of Magnetic Field on MRG Capacitance

The results show that the voltage across the MRG showed a slow first-order system characteristic when a step input voltage was applied. The electrical response of the MRG seemed to have a similar response to a capacitor. Thus, the capacitance of MRG under the effect of magnetic field was investigated. A step input of 1 V DC was applied to the circuit and the voltage across the MRG sample over time under various magnetic fields was recorded. For each test, the voltage across the MRG sample was measured for more than 30 min, which allowed enough time to reach the steady state. The capacitance was calculated from the rising time of the voltage measurement.

**Figure 4:**
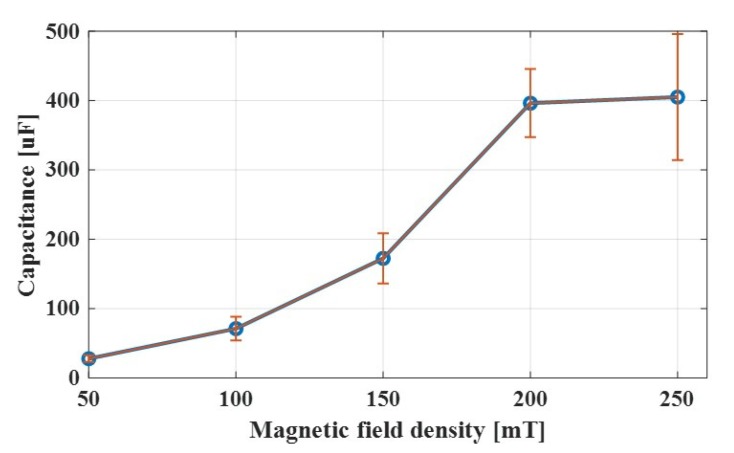
Variation in MRG capacitance with magnetic field density.

The capacitance of MRG was observed to be increasing as stronger magnetic fields were applied, as shown in Figure 4. As the iron particles became more chain-like, it created more regular patterns of iron particles sandwiched by base matrix. This would probably have a higher capacitance than irregular patterns of iron particles and base matrix.

### 3.4. Repeatability of MRG Resistance under DC Voltage

Since the CIPs in MRGs could move slowly through the aqua gelatin matrix and other environment factors such as dehydration could alter the nominal property of the material, it was necessary to investigate the repeatability of MRG resistance before the material was irreversibly modified.

The results in Figure 2 show that, when a step input voltage was applied, it required some time to settle to a stable state. Therefore, throughout the experiments, the anisotropic MRG samples were initially set at a stable value of resistance by applying a 1 V DC voltage for some time.

**Figure 5:**
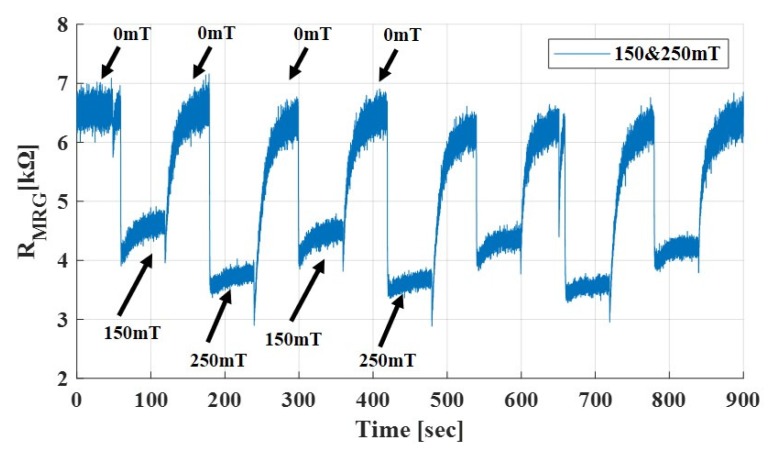
Repeatability of resistance change by applying 150 mT and 250 mT in sequence.

To see repeatability of MRG resistance, magnetic field was applied on and off periodically. In addition, the magnitude of magnetic field applied was 150 mT and 250 mT, alternatively. The resistance of MRG was measured and recorded, as shown in Figure 5.

The results show that the repeatability of MRG resistance due to external magnetic field was within acceptable error. However, for long-term application, the material property could be altered due to dehydration and other environment factors, which could degrade the sensing performance of the material. For further work, it is necessary to make the material more stable to enhance the repeatability performance.

## 4. Effect of Magnetic Field on MRG Impedance Change under AC Input Voltage

### 4.1. MRG Impedance under a Constant AC Frequency

**Figure 6:**
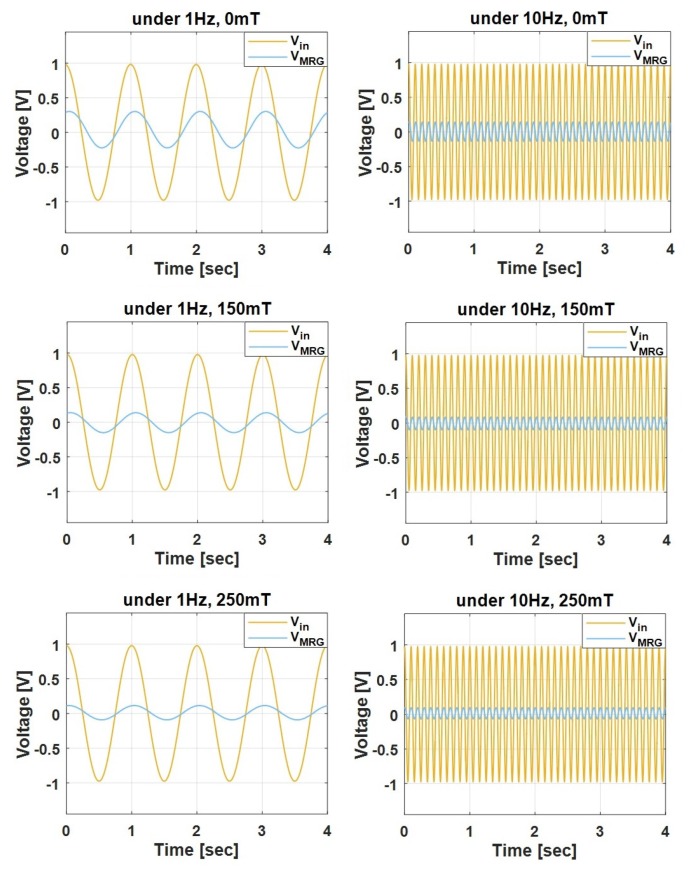
Measurement results of AC input voltage and the voltage measured at MRG in the time domain.

The response of MRG impedance change by magnetic field with the input AC voltage was investigated. A sinusoidal signal with a peak amplitude of 1 V was supplied to the MRG using the test circuit shown in Figure 1. First, the frequency of the AC voltage was set at 1 Hz. Then, the experiment was repeated for input AC voltage of 10 Hz. The magnetic field density was varied from 0 to 250 mT at 50 mT intervals and the changes of the magnitude and phase of MRG impedance were analyzed, as shown in Figure 6 and in Table 1.

### 4.2. MRG Impedance under Various AC Frequency

The frequency of the voltage was controlled as 0.1, 0.2, 0.5, 1, 2, and 5 Hz, as well as 10–100 Hz at 10 Hz intervals. For each frequency, the magnetic field density was varied from 0 to 250 mT at 50 mT intervals. The magnitudes of MRG impedance were calculated under different magnetic field densities and the input voltage frequencies.

**Figure 7:**
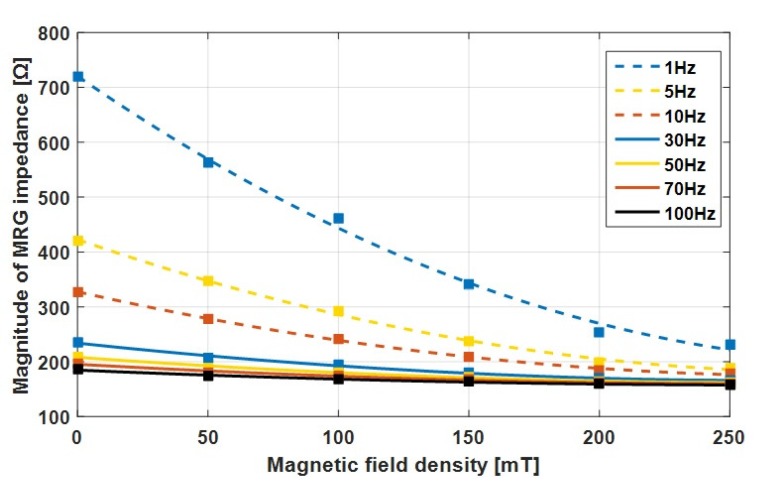
Variation in the MRG impedance magnitude and quadratic equation curve fitted results with magnetic field density for an MRG magnetized at 150 mT.

The changes in the MRG impedance by the magnetic field density at each input voltage frequency are plotted in Figure 7. The results show that the changes of the MRG impedance have unique graphs for each input frequency.

The relationship of the magnitude of MRG impedance ($\left|Z_{MRG}\right|\left[\Omega \right]$) with respect to the applied magnetic flux density (B[T]), for each applied frequency, could be approximated by a quadratic function, as shown in Figure 6. The coefficients of the approximated function for each frequency are summarized in Table 2, where B is the applied magnetic field.

(2) $$\left|Z_{MRG}\right|=a_{2}B^{2}+a_{1}B+a_{0}$$

From these curves, the magnetic field density applied to the MRG could be sensed by measuring the magnitude of the MRG impedance. The results show that the impedance magnitude decreased gradually with an increase of the external magnetic field density. At lower frequencies of the input voltage, the magnitude of the MRG impedance was more sensitive to changes in the magnetic field. When the input voltage frequency was increased more than 50 Hz, the rate of impedance change was a low value. Thus, it was preferable to use a low frequency for the input voltage to have a large sensitivity in the magnetic field measurement.

**Figure 8:**
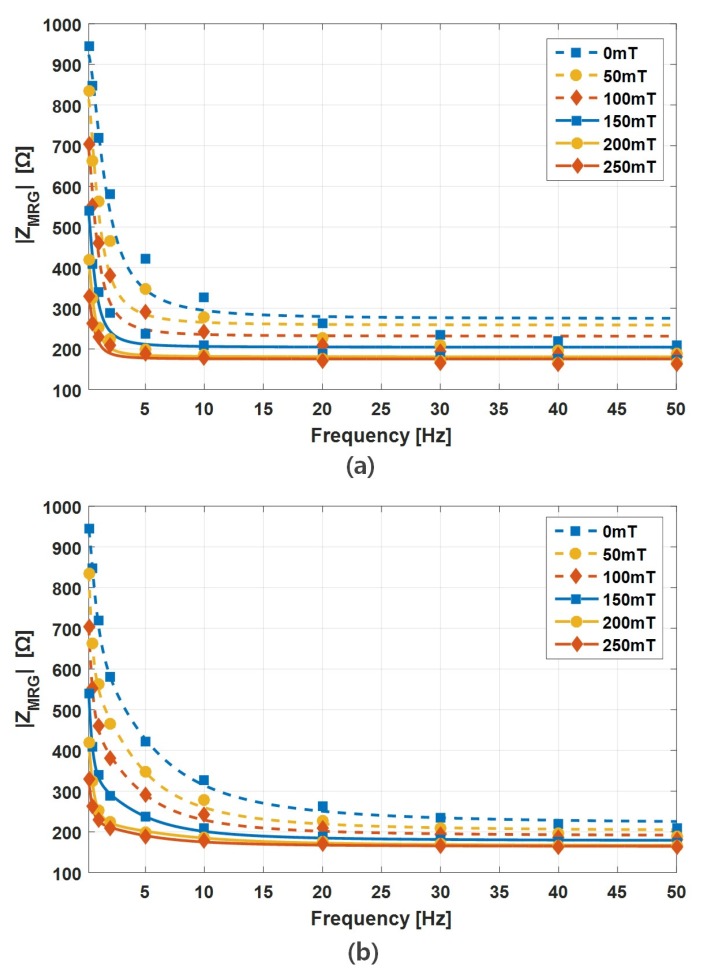
Variation in the MRG impedance magnitude and curve fitted results with voltage frequency for: (**a**) $Z_{MRG}=R_{0}+(R_{1}\left|\right|C_{1})$; and (**b**) $Z_{MRG}=R_{0}+(R_{1}\left|\right|C_{1})+(R_{2}\left|\right|C_{2})$.

The changes in the magnitude of impedance with respect to the input frequencies are plotted in Figure 8. With an increase of the input voltage frequency, under a constant magnetic field, the impedance decreased exponentially. The impedance magnitude decreased by as high as about 850 Ω when the input voltage frequency increased from 0.1 to 50 Hz under a constant magnetic field ranging from 0 to 250 mT. The rate of impedance change became negligible at higher than 40 Hz. For a stronger magnetic field, the impedance change was negligible at 20 Hz or higher. With a higher applied magnetic field, the rates of magnitude impedance also increased.

Based on experiment results, an electrical model for the MRG could be constructed as a system composed of resistor and capacitor. To generate the MRG electrical model, two different models (first-order system and second-order system) were assumed and analyzed.

**Figure 9:**
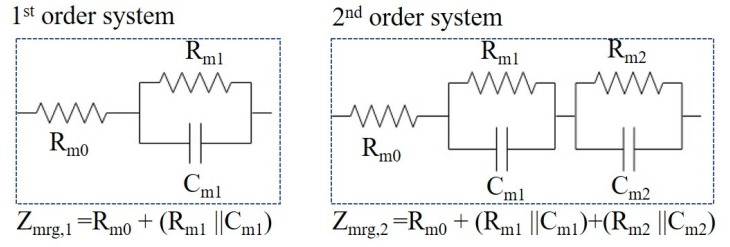
MRG impedance modeling with assumption of first-order system and second-order system.

When the MRG was assumed as a first-order system, as shown in Figure 9, it could be modeled as $Z_{MRG}=R_{m0}+(R_{m1}\left|\right|C_{m1})$ that derives the following modeling.

(3) $$Z_{MRG}=R_{m0}+\frac{R_{m1}}{1+j\omega R_{m1}C_{m1}}=\frac{R_{m0}+R_{m1}+j\omega R_{m0}R_{m1}C_{m1}}{1+j\omega R_{m1}C_{m1}}$$

Based on this model, the curve fit of the experiment data is shown in Figure 8a and the values of resistances and capacitance are summarized in Table 3.

The MRG was initially regarded as a simple model of a resistor and a capacitor in parallel, but the test data showed large deviations at low frequency region, indicating that the actual electrical modeling is a more complex system.

Thus, the MRG was modeled as a second-order system, composed of two series sets of a resistor and a capacitor in parallel, as shown in Figure 9. Then, the impedance of the MRG is composed of $Z_{MRG}=R_{m0}+(R_{m1}\left|\right|C_{m1})+(R_{m2}\left|\right|C_{m2})$ that results the following modeling.

(4) $$\begin{array}{c} Z_{MRG}=R_{m0}+\frac{R_{m1}}{1+j\omega R_{m1}C_{m1}}+\frac{R_{m2}}{1+j\omega R_{m2}C_{m2}}\\ =\frac{R_{m0}+R_{m1}+R_{m2}-\omega^{2}R_{m0}R_{m1}R_{m2}C_{m1}C_{m2}}{1-\omega^{2}R_{m0}R_{m1}R_{m2}C_{m1}C_{m2}+j\omega \left(R_{m1}C_{m1}+R_{m2}C_{m2}\right)}\\ +j\omega \frac{R_{m1}R_{m2}\left(C_{m1}+C_{m2}\right)+R_{m0}\left(R_{m1}C_{m1}+R_{m2}C_{m2}\right)}{1-\omega^{2}R_{m0}R_{m1}R_{m2}C_{m1}C_{m2}+j\omega \left(R_{m1}C_{m1}+R_{m2}C_{m2}\right)} \end{array}$$

**Figure 10:**
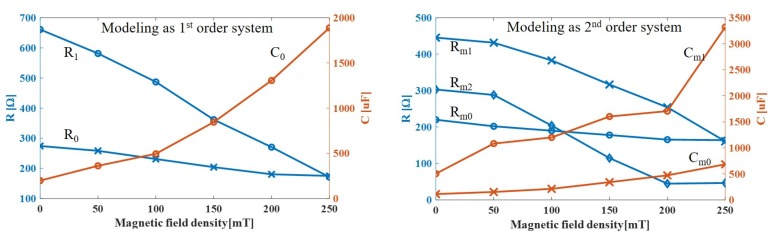
Variation of resistance and capacitance with respect to applied magnetic field density in the estimated model.

The curve fit of the data using this second-order system model is shown in Figure 8b and the values of resistances and capacitance are summarized in Table 4 and in Figure 10. It can be seen that, as the magnetic field increased, the resistors decreased while the capacitance increased, as we obtained a similar pattern with DC input voltage. This model is highly correlated with test data, for all frequency and magnetic field inputs.

Table 4 showd that, as the applied magnetic field increased, the resistances decreased while the capacitance increased, in accordance to the aforementioned experiment results.

In summary, the MRG can be modeled as a second-order system and the frequency of input voltage should be in a low value range to have a high resolution for detecting impedance change.

## 5. Conclusions

In this study, variable electric resistance and impedance of magnetorheological gel (MRG) due to external magnetic field were analyzed as a fundamental research for designing a magnetic flux sensor using that smart material. The changes of resistance and capacitance of MRG sample were measured under various magnetic fields with an input DC voltage. Experiment data showed that the changes of resistance decreased as the magnitude of magnetic field was increased. From the curve fitting of the data, a function of magnetic field and resistance model was derived. In addition, non-linear hysteresis of resistance change was observed with DC input voltage. When AC input voltage was applied, the impedance changes of the MRG sample became a function of both the magnetic field and the input voltage frequency. Experiment data showed that, as the frequency and magnetic field increased, the overall impedance decreased. The impedance due to magnetic field was modeled in frequency domain as a second-order system composed of multiple resistance and capacitance components. Although the elastic MRG sample contains a special property of resistance and impedance variation, which could be used as a sensor of magnetometer, there are some limitations that need to be overcome. First, there should be further studies on the behavior of MRG impedance with AC voltage input. In addition, there should be further study on modeling MRG impedance change, which is a complex and non-linear system with some hysteresis with advanced modeling tool such as deep neural network. Furthermore, studies on solving the hysteresis of the MRG by enhancing the material properties with additives should be conducted.

## Figures and Tables

**Table 1:** Magnitude and phase of MRG impedance for some selected input frequency.

		0	50 mT	100 mT	150 mT	200 mT	250 mT
1 Hz	$Z_{mag}\left(\Omega \right)$	579.8	465.4	380.2	287.9	225.2	209
	$Z_{phase}\left(deg\right)$	31.9	31.0	27.8	23.3	17.8	16.0
10 Hz	$Z_{mag}\left(\Omega \right)$	262.7	227.7	208.1	189.1	174.4	169.7
	$Z_{phase}\left(deg\right)$	28.9	26.0	20.8	13.9	8.7	7.3

**Table 2:** The quadratic equation curve fitting coefficients for the relationship between magnetic flux density and MRG impedance.

Voltage Freq.	$a_{2}$	$a_{1}$	$a_{0}$	Adjusted R-Square
1 Hz	5166	−3286	720.4	0.9934
5 Hz	2793	−1646	422.7	0.9967
10 Hz	1871	−1072	327.6	0.9977
30 Hz	941.8	−507.6	234	0.9920
50 Hz	628.5	−341.5	208.1	0.9823
70 Hz	495.1	−266.2	195.5	0.9791
100 Hz	369.6	−201	184.8	0.9777

**Table 3:** Curve fitting coefficients for the relationship between voltage frequency and MRG impedance when $Z_{MRG}=R_{0}+(R_{1}\left|\right|C_{1})$.

Magnetic Flux Density	$R_{0}$	$R_{1}$	$C_{1}$	Adjusted R-Square
0 mT	274.4	661.0	0.0002	0.9814
50 mT	258.4	581.5	0.0004	0.9543
100 mT	231.2	487.0	0.0005	0.9615
150 mT	204.2	362.0	0.0008	0.9663
200 mT	180.6	270.6	0.0013	0.9856
250 mT	175.2	171.2	0.0019	0.9599

**Table 4:** Curve fitting coefficients for the relationship between voltage frequency and MRG impedance when $Z_{MRG}=R_{m0}+(R_{m1}\left|\right|C_{m1})+(R_{m2}\left|\right|C_{m2})$.

Magnetic Flux Density	$R_{m0}$	$R_{m1}$	$R_{m2}$	$C_{m1}$	$C_{m2}$	Adjusted R-Square
0 mT	219.0	445.4	302.9	0.0005	0.0001	0.9993
50 mT	202.0	431.5	288.1	0.0011	0.0002	0.9985
100 mT	189.9	382.9	204.0	0.0012	0.0002	0.9986
150 mT	178.0	316.7	114.7	0.0016	0.0003	0.9988
200 mT	165.4	254.2	44.59	0.0017	0.0005	0.9996
250 mT	163.9	160.7	46.45	0.0033	0.0007	0.9983
